# Supplementary material for: Pure oxygen ventilation during general anaesthesia does not result in increased postoperative respiratory morbidity but decreases surgical site infection. An observational clinical study
Source: PeerJ. 2014 Oct 9;2:e613. doi: 10.7717/peerj.613 (PMC4194458; doi:10.7717/peerj.613)
Supplement: Supplemental Information 1 [file peerj-02-613-s001.pdf]

Postoperative Hypoxia (%); **1995**: All Patients with N_2O (70%) + O_2 (30%); **1996** changing regimen; from **1997** all patients with FiO_2 = 1.0

HYP OXIA (%)	ALL	General Surgery				Gynecology				Orthopedic Surgery				Vascular Surgery				
	76,784	ALL	Minor	Major	Colon	ALL	Minor	Major	Mamma	All	Minor	Major	Spine	All	Minor	Aorta	Peripher	Carotid
1995	5313	1322	765	231	326	779	510	189	80	1769	997	693	79	1443	342	271	630	200
	4.3	3.9	4.1	3.5	3.4	2.3	0.8	5.8	3.8	4.5	4.3	4.6	6.3	5.4	3.2	8.1	6.5	2.0
1996	5079	1123	656	245	222	739	418	212	109	1747	1021	641	85	1470	383	290	574	223
	4.4	4.5	4.7	4.5	4.1	3.0	1.2	5.7	4.6	4.6	4.4	4.8	5.9	4.9	3.1	7.2	6.3	1.3
1997	5245	1351	838	220	293	736	471	190	75	1749	990	656	103	1409	350	244	620	195
	2.9	2.7	2.7	2.3	2.7	2.2	0.6	4.2	4.0	2.6	2.7	2.3	3.9	4.2	2.6	6.1	5.5	0.5
1998	4830	1185	663	241	281	746	443	188	55	1650	902	650	98	1249	411	247	390	201
	2.6	2.4	2.6	2.1	2.5	1.2	0.5	3.7	0	2.5	2.4	2.5	3.1	3.7	2.9	4.5	5.6	0.5
1999	4894	1044	609	214	221	946	593	235	118	1752	925	718	109	1152	355	189	435	173
	2.3	2.3	2.5	1.9	2.3	1.1	0.2	3.4	0.8	2.0	2.1	1.9	1.8	3.8	3.1	3.7	6.0	0
2000	4850	1054	694	171	189	936	604	183	149	1772	952	713	107	1088	346	156	419	167
	2.2	2.1	2.3	1.8	1.6	0.9	0.3	2.7	0.7	1.9	2.2	1.5	1.9	4.1	4.0	5.1	5.5	0
2001	4782	1015	672	160	183	915	581	201	133	1739	933	705	101	1113	342	173	406	192
	2.0	2.1	2.1	1.9	2.2	0.9	0.2	3.5	0	1.6	1.8	1.4	1.0	3.3	2.6	4.0	5.2	0
2002	5171	1501	885	314	302	1044	637	282	125	1708	855	728	125	918	267	98	383	170
	1.7	1.9	2.3	1.6	1.0	0.7	0.2	1.8	0.8	1.5	1.4	1.2	3.2	3.3	3.0	3.1	4.4	1.2
2003	5380	1551	804	391	356	981	594	268	119	1907	1058	719	130	941	323	107	353	158
	1.6	1.8	2.4	1.3	1.1	0.5	0	1.5	0.8	1.2	1.0	1.4	1.5	3.2	2.5	2.8	5.1	0.6
2004	5156	1512	841	375	296	867	524	214	129	1827	1061	677	89	950	341	151	302	156
	1.4	1.7	1.9	1.1	1.7	0.6	0	1.4	1.6	0.8	0.8	0.9	0	2.6	2.6	2.6	4.0	0
2005	5081	1443	785	358	300	893	539	205	149	1851	1005	724	122	894	305	164	307	118
	1.3	1.5	1.5	1.7	1.3	0.4	0	1.0	1.3	0.7	0.9	0.6	0	2.8	2.0	2.4	4.9	0
2006	5228	1447	751	334	362	876	547	165	164	1960	1031	767	162	945	263	132	430	120
	1.4	1.8	1.9	1.8	1.7	0.7	0	1.8	1.8	0.7	0.7	0.7	1.2	3.1	2.7	2.3	4.0	1.7
2007	5160	1373	703	319	351	805	483	155	167	2092	1276	690	126	890	253	107	414	116
	1.1	1.5	1.6	1.6	1.4	0.5	0.6	0	0.6	0.6	0.5	0.9	0.8	2.4	2.4	3.7	2.7	0
2008	5403	1609	805	418	386	830	539	146	145	2071	1199	752	120	893	349	110	332	102
	1.1	1.7	2.0	1.7	1.3	0.4	0.4	0.7	0	0.7	0.6	0.9	0	1.9	1.4	2.7	2.7	0
2009	5212	1584	820	441	323	827	478	164	185	1876	1028	735	113	925	279	131	396	119
	1.0	1.5	1.7	1.4	0.9	0.2	0	0.6	0.5	0.5	0.4	0.7	0	1.7	1.4	3.1	2.0	0
