# Supplementary material for: Pure oxygen ventilation during general anaesthesia does not result in increased postoperative respiratory morbidity but decreases surgical site infection. An observational clinical study
Source: PeerJ. 2014 Oct 9;2:e613. doi: 10.7717/peerj.613 (PMC4194458; doi:10.7717/peerj.613)
Supplement: Supplemental Information 3 [file peerj-02-613-s003.pdf]

In-Hospital-MORTALITY (%); **1995**: All Patients with N_2O (70%) + O_2 (30%); **1996** changing regimen; from **1997** all patients with FiO_2 = 1.0

MOR TAL ITY (%)	ALL	General Surgery				Gynecology				Orthopedic Surgery				Vascular Surgery				
	76,784	ALL	Minor	Major	Colon	ALL	Minor	Major	Mamma	All	Minor	Major	Spine	**All**	Minor	Aorta	Peripher	Carotid
1995	5313	**1322**	765	231	326	**779**	510	189	80	**1769**	997	693	79	**1443**	342	271	630	200
	**2.1**	**2.5**	**0.4**	**6.9**	**4.3**	**0.8**	**0.2**	**1.6**	**2.5**	**0.5**	**0.2**	**0.7**	**2.5**	**4.5**	**0**	**5.2**	**7.3**	**2.5**
1996	5079	**1123**	656	245	222	**739**	418	212	109	**1747**	1021	641	85	**1470**	383	290	574	223
	**1.8**	**2.5**	**0.3**	**6.1**	**5.0**	**0.8**	**0.2**	**1.9**	**0.9**	**0.4**	**0.1**	**0.9**	**0**	**3.5**	**0**	**5.2**	**6.1**	**0.9**
1997	5245	**1351**	838	220	293	**736**	471	190	75	**1749**	990	656	103	**1409**	350	244	620	195
	**1.6**	**1.9**	**0.5**	**4.1**	**4.1**	**0.4**	**0.2**	**1.1**	**0**	**0.4**	**0.2**	**0.6**	**1.0**	**3.4**	**0**	**4.5**	**5.5**	**1.5**
1998	4830	**1185**	663	241	281	**746**	443	188	55	**1650**	902	650	98	**1249**	411	247	390	201
	**1.4**	**2.1**	**0.5**	**4.6**	**3.9**	**0.4**	**0**	**1.1**	**1.8**	**0.3**	**0**	**0.8**	**0**	**2.7**	**0**	**3.2**	**6.4**	**0.5**
1999	4894	**1044**	609	214	221	**946**	593	235	118	**1752**	925	718	109	**1152**	355	189	435	173
	**1.3**	**1.6**	**0.3**	**6.5**	**4.5**	**0.3**	**0**	**1.3**	**0**	**0.2**	**0**	**0.6**	**0**	**3.4**	**0.3**	**2.1**	**7.6**	**0.6**
2000	4850	**1054**	694	171	189	**936**	604	183	149	**1772**	952	713	107	**1088**	346	156	419	167
	**1.3**	**2.2**	**0.1**	**7.0**	**5.3**	**0.3**	**0**	**1.6**	**0**	**0.2**	**0**	**0.6**	**0**	**2.8**	**0**	**1.9**	**6.2**	**1.2**
2001	4782	**1015**	672	160	183	**915**	581	201	133	**1739**	933	705	101	**1113**	342	173	406	192
	**1.2**	**1.9**	**0**	**6.9**	**4.4**	**0.3**	**0**	**1.0**	**0.8**	**0.2**	**0.1**	**0.4**	**0**	**2.4**	**0**	**2.3**	**5.7**	**0**
2002	5171	**1501**	885	314	302	**1044**	637	282	125	**1708**	855	728	125	**918**	267	98	383	170
	**1.3**	**2.3**	**0.2**	**6.7**	**3.6**	**0.4**	**0.2**	**0.7**	**0.8**	**0.2**	**0**	**0.3**	**0.8**	**2.6**	**0.4**	**3.1**	**5.5**	**0**
2003	5380	**1551**	804	391	356	**981**	594	268	119	**1907**	1058	719	130	**941**	323	107	353	158
	**1.4**	**2.6**	**0.1**	**6.4**	**3.9**	**0.5**	**0.3**	**1.1**	**0**	**0.2**	**0.2**	**0.1**	**0.8**	**2.8**	**0**	**3.7**	**5.7**	**1.3**
2004	5156	**1512**	841	375	296	**867**	524	214	129	**1827**	1061	677	89	**950**	341	151	302	156
	**1.1**	**2.1**	**0**	**5.9**	**3.0**	**0.3**	**0**	**1.4**	**0**	**0**	**0**	**0**	**0**	**2.6**	**0.6**	**2.6**	**6.0**	**0.6**
2005	5081	**1443**	785	358	300	**893**	539	205	149	**1851**	1005	724	122	**894**	305	164	307	118
	**1.2**	**1.9**	**0**	**5.3**	**2.7**	**0.6**	**0.2**	**1.5**	**1.3**	**0.05**	**0**	**0.1**	**0**	**2.9**	**0**	**1.8**	**6.8**	**1.7**
2006	5228	**1447**	751	334	362	**876**	547	165	164	**1960**	1031	767	162	**945**	263	132	430	120
	**1.4**	**2.4**	**0**	**6.0**	**4.1**	**0.3**	**0**	**1.2**	**0.6**	**0.2**	**0**	**0.3**	**1.2**	**3.1**	**0.4**	**3.0**	**5.6**	**0**
2007	5160	**1373**	703	319	351	**805**	483	155	167	**2092**	1276	690	126	**890**	253	107	414	116
	**1.3**	**2.3**	**0.1**	**5.3**	**4.0**	**0.4**	**0**	**1.3**	**0.6**	**0.1**	**0.08**	**0.1**	**0.8**	**3.1**	**0**	**0.9**	**6.5**	**0**
2008	5403	**1609**	805	418	386	**830**	539	146	145	**2071**	1199	752	120	**893**	349	110	332	102
	**1.3**	**2.9**	**0.2**	**6.5**	**4.4**	**0.5**	**0.2**	**2.1**	**0**	**0**	**0**	**0**	**0**	**2.4**	**0.3**	**0**	**5.7**	**1.0**
2009	5212	**1584**	820	441	323	**827**	478	164	185	**1876**	1028	735	113	**925**	279	131	396	119
	**1.1**	**2.2**	**0.1**	**5.0**	**3.7**	**0.4**	**0**	**1.2**	**0.5**	**0**	**0**	**0**	**0**	**2.1**	**0**	**0.8**	**4.3**	**0.8**
