# Supplementary material for: Pure oxygen ventilation during general anaesthesia does not result in increased postoperative respiratory morbidity but decreases surgical site infection. An observational clinical study
Source: PeerJ. 2014 Oct 9;2:e613. doi: 10.7717/peerj.613 (PMC4194458; doi:10.7717/peerj.613)
Supplement: Supplemental Information 5 [file peerj-02-613-s005.pdf]

PONV (%); **1995**: All Patients with N_2O (70%) + O_2 (30%); **1996** changing regimen; from **1997** all patients with FiO_2 = 1.0

PONV (%)	ALL	General Surgery				Gynecology				Orthopedic Surgery				Vascular Surgery				
	76,784	ALL	Minor	Major	Colon	ALL	Minor	Major	Mamma	All	Minor	Major	Spine	**All**	Minor	Aorta	Peripher	Carotid
1995	5313	**1322**	765	231	326	**779**	510	189	80	**1769**	997	693	79	**1443**	342	271	630	200
	**21.6**	**23.5**	**24.2**	**25.6**	**20.6**	**21.3**	**21.4**	**20.1**	**23.8**	**20.6**	**20.2**	**20.5**	**31.6**	**21.3**	**17.0**	**18.1**	**25.1**	**21.5**
1996	5079	**1123**	656	245	222	**739**	418	212	109	**1747**	1021	641	85	**1470**	383	290	574	223
	**19.5**	**20.1**	**20.0**	**20.8**	**19.8**	**17.8**	**17.7**	**17.0**	**21.1**	**19.7**	**18.5**	**20.6**	**28.2**	**19.5**	**14.6**	**16.2**	**24.0**	**20.6**
1997	5245	**1351**	838	220	293	**736**	471	190	75	**1749**	990	656	103	**1409**	350	244	620	195
	**17.5**	**19.0**	**19.0**	**20.9**	**17.7**	**16.8**	**17.6**	**14.2**	**18.7**	**16.5**	**15.8**	**16.9**	**21.4**	**17.5**	**13.4**	**14.8**	**20.0**	**18.5**
1998	4830	**1185**	663	241	281	**746**	443	188	55	**1650**	902	650	98	**1249**	411	247	390	201
	**16.8**	**18.6**	**19.3**	**17.4**	**17.8**	**15.0**	**16.9**	**13.3**	**21.8**	**16.4**	**15.6**	**16.6**	**22.4**	**16.7**	**13.4**	**15.0**	**20**	**18.9**
1999	4894	**1044**	609	214	221	**946**	593	235	118	**1752**	925	718	109	**1152**	355	189	435	173
	**16.9**	**19.4**	**19.5**	**21.0**	**17.6**	**16.7**	**17.2**	**13.2**	**21.2**	**15.8**	**15.1**	**16.3**	**17.4**	**16.4**	**12.4**	**15.3**	**19.1**	**19.1**
2000	4850	**1054**	694	171	189	**936**	604	183	149	**1772**	952	713	107	**1088**	346	156	419	167
	**17.0**	**18.0**	**18.2**	**19.3**	**16.4**	**17.8**	**19.0**	**13.1**	**18.8**	**15.9**	**15.0**	**17.0**	**16.8**	**16.8**	**13.0**	**14.7**	**19.3**	**20.4**
2001	4782	**1015**	672	160	183	**915**	581	201	133	**1739**	933	705	101	**1113**	342	173	406	192
	**17.1**	**18.8**	**18.6**	**19.4**	**19.1**	**16.6**	**17.0**	**13.4**	**19.5**	**15.9**	**14.9**	**16.9**	**17.8**	**17.4**	**13.7**	**17.3**	**19.5**	**19.8**
2002	5171	**1501**	885	314	302	**1044**	637	282	125	**1708**	855	728	125	**918**	267	98	383	170
	**17.0**	**18.5**	**18.2**	**20.7**	**17.2**	**16.2**	**17.4**	**12.4**	**18.4**	**16.0**	**14.9**	**16.9**	**18.4**	**17.5**	**12.0**	**16.3**	**20.1**	**21.2**
2003	5380	**1551**	804	391	356	**981**	594	268	119	**1907**	1058	719	130	**941**	323	107	353	158
	**16.7**	**17.9**	**18.3**	**18.2**	**16.6**	**15.9**	**17.2**	**12.3**	**17.6**	**15.9**	**15.4**	**16.7**	**16.2**	**17.2**	**11.5**	**15.9**	**21.2**	**20.9**
2004	5156	**1512**	841	375	296	**867**	524	214	129	**1827**	1061	677	89	**950**	341	151	302	156
	**16.6**	**17.7**	**17.8**	**18.7**	**16.2**	**17.0**	**18.7**	**12.1**	**17.8**	**16.3**	**15.6**	**16.8**	**19.0**	**15.9**	**10.3**	**15.9**	**20.9**	**18.6**
2005	5081	**1443**	785	358	300	**893**	539	205	149	**1851**	1005	724	122	**894**	305	164	307	118
	**16.3**	**17.3**	**17.6**	**16.5**	**17.7**	**15.3**	**16.5**	**10.7**	**17.4**	**16.4**	**15.5**	**17.4**	**17.2**	**15.8**	**9.8**	**23.0**	**21.5**	**18.6**
2006	5228	**1447**	751	334	362	**876**	547	165	164	**1960**	1031	767	162	**945**	263	132	430	120
	**16.2**	**17.4**	**17.7**	**17.0**	**16.9**	**15.5**	**16.1**	**11.5**	**17.7**	**16.1**	**15.0**	**17.0**	**18.5**	**15.4**	**9.9**	**15.9**	**18.6**	**15.8**
2007	5160	**1373**	703	319	351	**805**	483	155	167	**2092**	1276	690	126	**890**	253	107	414	116
	**15.5**	**17.0**	**17.8**	**17.6**	**15.1**	**15.8**	**16.4**	**11.0**	**18.6**	**14.6**	**12.9**	**17.2**	**17.5**	**15.3**	**8.7**	**15.0**	**19.6**	**14.7**
2008	5403	**1609**	805	418	386	**830**	539	146	145	**2071**	1199	752	120	**893**	349	110	332	102
	**15.3**	**17.2**	**17.5**	**17.7**	**15.8**	**14.7**	**15.0**	**11.6**	**16.6**	**14.5**	**13.3**	**16.4**	**15.8**	**14.1**	**8.9**	**13.6**	**19.0**	**16.7**
2009	5212	**1584**	820	441	323	**827**	478	164	185	**1876**	1028	735	113	**925**	279	131	396	119
	**15.4**	**17.0**	**17.0**	**17.5**	**16.4**	**15.5**	**16.1**	**11.0**	**17.8**	**14.9**	**14.7**	**15.1**	**15.0**	**13.5**	**9.0**	**12.9**	**16.4**	**15.1**
