# Supplementary material for: Pure oxygen ventilation during general anaesthesia does not result in increased postoperative respiratory morbidity but decreases surgical site infection. An observational clinical study
Source: PeerJ. 2014 Oct 9;2:e613. doi: 10.7717/peerj.613 (PMC4194458; doi:10.7717/peerj.613)
Supplement: Supplemental Information 6 [file peerj-02-613-s006.pdf]

PONV (N); **1995**: All Patients with N_2O (70%) + O_2 (30%); **1996** changing regimen; from **1997** all patients with FiO_2 = 1.0

PONV (N)	ALL	General Surgery				Gynecology				Orthopedic Surgery				Vascular Surgery				
	76,784	ALL	Minor	Major	Colon	ALL	Minor	Major	Mamma	All	Minor	Major	Spine	All	Minor	Aorta	Peripher	Carotid
1995	5313	**1322**	765	231	326	**779**	510	189	80	**1769**	997	693	79	**1443**	342	271	630	200
	**1149**	**311**	**185**	**59**	**67**	**166**	**109**	**38**	**19**	**364**	**201**	**142**	**25**	**308**	**58**	**49**	**158**	**43**
1996	5079	**1123**	656	245	222	**739**	418	212	109	**1747**	1021	641	85	**1470**	383	290	574	223
	**991**	**226**	**131**	**51**	**44**	**133**	**74**	**36**	**23**	**345**	**189**	**132**	**24**	**287**	**56**	**47**	**138**	**46**
1997	5245	**1351**	838	220	293	**736**	471	190	75	**1749**	990	656	103	**1409**	350	244	620	195
	**916**	**257**	**159**	**46**	**52**	**124**	**83**	**27**	**14**	**289**	**156**	**111**	**22**	**246**	**47**	**36**	**127**	**36**
1998	4830	**1185**	663	241	281	**746**	443	188	55	**1650**	902	650	98	**1249**	411	247	390	201
	**811**	**220**	**128**	**42**	**50**	**112**	**75**	**25**	**12**	**271**	**141**	**108**	**22**	**208**	**55**	**37**	**78**	**38**
1999	4894	**1044**	609	214	221	**946**	593	235	118	**1752**	925	718	109	**1152**	355	189	435	173
	**826**	**203**	**119**	**45**	**39**	**158**	**102**	**31**	**25**	**276**	**140**	**117**	**19**	**189**	**44**	**29**	**83**	**33**
2000	4850	**1054**	694	171	189	**936**	604	183	149	**1772**	952	713	107	**1088**	346	156	419	167
	**822**	**190**	**126**	**33**	**31**	**167**	**115**	**24**	**28**	**282**	**143**	**121**	**18**	**183**	**45**	**23**	**81**	**34**
2001	4782	**1015**	672	160	183	**915**	581	201	133	**1739**	933	705	101	**1113**	342	173	406	192
	**813**	**191**	**125**	**31**	**35**	**152**	**99**	**27**	**26**	**276**	**139**	**119**	**18**	**194**	**47**	**30**	**79**	**38**
2002	5171	**1501**	885	314	302	**1044**	637	282	125	**1708**	855	728	125	**918**	267	98	383	170
	**881**	**278**	**161**	**65**	**52**	**169**	**111**	**35**	**23**	**273**	**127**	**123**	**23**	**161**	**32**	**16**	**77**	**36**
2003	5380	**1551**	804	391	356	**981**	594	268	119	**1907**	1058	719	130	**941**	323	107	353	158
	**899**	**277**	**147**	**71**	**59**	**156**	**102**	**33**	**21**	**304**	**163**	**120**	**21**	**162**	**37**	**17**	**75**	**33**
2004	5156	**1512**	841	375	296	**867**	524	214	129	**1827**	1061	677	89	**950**	341	151	302	156
	**863**	**268**	**150**	**70**	**48**	**147**	**98**	**26**	**23**	**297**	**166**	**114**	**17**	**151**	**35**	**24**	**63**	**29**
2005	5081	**1443**	785	358	300	**893**	539	205	149	**1851**	1005	724	122	**894**	305	164	307	118
	**831**	**250**	**138**	**59**	**53**	**137**	**89**	**22**	**26**	**303**	**156**	**126**	**21**	**141**	**30**	**23**	**66**	**22**
2006	5228	**1447**	751	334	362	**876**	547	165	164	**1960**	1031	767	162	**945**	263	132	430	120
	**849**	**251**	**133**	**57**	**61**	**136**	**88**	**19**	**29**	**316**	**155**	**131**	**30**	**146**	**26**	**21**	**80**	**19**
2007	5160	**1373**	703	319	351	**805**	483	155	167	**2092**	1276	690	126	**890**	253	107	414	116
	**802**	**234**	**125**	**56**	**53**	**127**	**79**	**17**	**31**	**305**	**164**	**119**	**22**	**136**	**22**	**16**	**81**	**17**
2008	5403	**1609**	805	418	386	**830**	539	146	145	**2071**	1199	752	120	**893**	349	110	332	102
	**825**	**276**	**141**	**74**	**61**	**122**	**81**	**17**	**24**	**301**	**159**	**123**	**19**	**126**	**31**	**15**	**63**	**17**
2009	5212	**1584**	820	441	323	**827**	478	164	185	**1876**	1028	735	113	**925**	279	131	396	119

	801	269	139	77	53	128	77	18	33	279	151	111	17	125	25	17	65	18
