# Supplementary material for: Pure oxygen ventilation during general anaesthesia does not result in increased postoperative respiratory morbidity but decreases surgical site infection. An observational clinical study
Source: PeerJ. 2014 Oct 9;2:e613. doi: 10.7717/peerj.613 (PMC4194458; doi:10.7717/peerj.613)
Supplement: Supplemental Information 12 [file peerj-02-613-s012.pdf]

Surgical procedures from 1995 to 2009 (GS = general surgery; VS = vascular surgery)

OP –[N /%]	1995	1996	1997	1998	1999	2000	2001	2002	2003	2004	2005	2006	2007	2008	2009
**ALL - 76,784/100**	**5313**	**5079**	**5245**	**4830**	**4894**	**4850**	**4782**	**5171**	**5380**	**5156**	**5081**	**5228**	**5160**	**5403**	**5212**
**GS - 20,114/26.2**	**1322**	**1123**	**1351**	**1185**	**1044**	**1054**	**1015**	**1501**	**1551**	**1512**	**1443**	**1447**	**1373**	**1609**	**1584**
Colorectal	326/24.7	222/19.8	293/21.7	281/23.7	221/21.2	189/17.9	183/18.0	302/20.1	356/23.0	296/19.6	300/20.8	362/25.0	351/25.6	386/24.0	323/20.4
Small bowel	124/9.4	132/11.8	116/8.6	120/10.1	102/9.8	97/9.2	95/9.4	160/10.7	184/11.9	140/9.6	145/10.0	103/7.1	62/4.5	94/5.8	107/6.8
Gastrectomy	29/2.2	55/4.9	35/2.6	50/4.2	53/5.1	29/2.8	30/3.0	57/3.8	65/4.2	84/5.6	82/5.7	66/4.6	93/6.8	105/6.5	99/6.3
Esophag, Whip-ple	24/1.8	31/2.8	29/2.1	17/1.4	18/1.7	17/1.6	12/1.2	28/1.9	31/2.0	47/3.1	36/2.5	56/3.9	65/4.7	86/5.3	78/4.9
Hepatectomy	29/2.2	10/0.9	17/1.3	35/3.0	23/2.2	14/1.3	12/1.2	37/2.5	49/3.2	56/3.7	51/3.5	54/3.7	58/4.2	78/4.8	91/5.7
Lung resection	25/1.8	17/1.5	23/1.7	19/1.6	18/1.7	14/1.3	11/1.1	32/2.1	62/4.0	48/3.2	44/3.0	55/3.8	41/3.0	55/3.4	66/4.2
CHE, App., mi-nor	516/39.0	499/44.4	498/36.9	446/376	406/38.9	456/43.3	455/44.8	567/37.8	527/34.0	611/40.4	534/37.0	492/34.0	406/29.6	537/33.4	521/32.9
Hernia, Struma	249/18.9	157/13.9	340/25.1	217/18.4	203/19.4	238/22.6	217/21.7	318/21.1	277/17.7	230/15.1	251/17.5	259/17.9	297/21.6	268/16.8	299/18.8
**GYN - 12,920/16.8**	**779**	**739**	**736**	**746**	**946**	**936**	**915**	**1044**	**981**	**867**	**893**	**876**	**805**	**830**	**827**
AUE, Lap, Vulva	82/10.5	91/12.3	89/12.1	101/13.5	135/14.3	91/9.7	108/11.8	169/16.2	181/18.5	146/16.8	144/16.1	116/13.2	118/14.7	104/12.5	127/15.3
Cesarean Sect	107/13.7	121/16.4	101/13.7	87/11.7	100/10.6	92/9.8	93/10.2	113/10.8	87/8.7	68/7.8	61/6.8	49/5.6	37/4.6	42/5.1	37/4.5
Major Breast	80/10.3	109/14.7	75/10.2	55/7.3	118/12.5	149/15.9	133/14.5	125/12.0	119/12.1	129/14.9	149/16.7	164/18.7	167/20.7	145/17.5	185/22.4
LSK, Abr, minor	434/55.7	347/47.0	382/51.9	372/49.9	534/56.4	532/56.8	501/54.8	554/531	498/50.8	467/53.9	477/53.4	478/54.6	422/52.4	482/58.0	386/46.7
VUE	76/9.8	71/9.6	89/12.1	71/9.5	59/6.2	72/7.8	80/8.7	83/7.9	96/9.9	57/6.6	62/7.0	69/7.9	61/7.6	57/6.9	92/11.1
**ORTH – 27,470/35.8**	**1769**	**1747**	**1749**	**1650**	**1752**	**1772**	**1739**	**1708**	**1907**	**1827**	**1851**	**1960**	**2092**	**2071**	**1876**
A'plasty, major	693/39.2	641/36.7	656/37.5	650/39.4	718/41.0	713/40.2	705/40.5	728/42.6	719/37.7	677/37.1	724/39.1	767/39.1	690/33.0	752/36.3	735/39.2
Major Spine	79/4.5	85/4.9	103/5.9	98/5.9	109/6.2	107/6.0	101/5.8	125/7.3	89/4.9	89/4.9	122/6.6	162/8.3	126/6.0	120/5.8	113/6.0
Nucl, Kyph, minor	997/56.3	1021/58.4	990/56.6	902/54.7	925/52.8	952/53.8	933/53.7	855/50.1	1061/58.0	1061/58.0	1005/54.3	1031/52.6	1276/61.0	1199/57.9	1028/54.8
**VS – 16,280/21.2**	**1443**	**1470**	**1409**	**1249**	**1152**	**1088**	**1113**	**918**	**941**	**950**	**894**	**945**	**890**	**893**	**925**
Aorta abdominal	247/17.1	262/17.8	222/15.8	219/17.5	163/14.2	132/12.1	153/13.7	87/9.5	98/10.4	137/14.4	157/17.6	122/12.9	92/10.3	98/11.0	115/12.4
Aorta thoracic	24/1.7	28/1.9	22/1.6	28/2.2	26/2.3	24/2.2	20/1.8	11/1.2	9/1.0	14/1.5	7/0.8	10/1.1	15/1.7	12/1.3	16/1.7
Revascularization	553/38.3	505/34.4	533/37.8	347/27.8	369/32.0	360/33.1	341/30.6	345/37.6	316/33.6	271/28.5	265/29.6	407/43.1	387/43.5	306/34.3	350/37.8
Cerebral artery	200/13.9	223/15.2	195/13.8	201/16.1	173/15.0	167/15.3	192/17.3	170/18.5	158/16.8	156/16.4	118/13.2	120/12.7	116/13.0	102/11.4	119/12.9
Thigh-amputation	77/5.3	69/4.7	87/6.2	43/3.4	66/5.7	59/5.4	65/5.8	38/4.1	37/3.9	31/3.3	42/4.7	23/2.4	27/3.0	26/2.9	46/5.0
Varices, shunt, PM	342/23.7	383/26.0	350/24.8	411/33.0	355/30.8	346/31.9	342/30.8	267/19.1	323/34.3	341/35.9	305/34.1	263/27.8	253/28.5	349/39.1	279/30.2
